# The SCI Exercise Self-Efficacy Scale (ESES): development and psychometric properties

**DOI:** 10.1186/1479-5868-4-34

**Published:** 2007-08-30

**Authors:** Thilo Kroll, Matthew Kehn, Pei-Shu Ho, Suzanne Groah

**Affiliations:** 1 University of Dundee, Alliance for Self Care Research, Department of Nursing & Midwifery, Dundee, Scotland, UK; 2 National Rehabilitation Hospital (NRH), Neuroscience Research Center, Washington, DC, USA; 3 Westat, Rockville, MD, USA

## Abstract

**Background:**

Rising prevalence of secondary conditions among persons with spinal cord injury (SCI) has focused recent attention to potential health promotion programs designed to reduce such adverse health conditions. A healthy lifestyle for people with SCI, including and specifically, the adoption of a vigorous exercise routine, has been shown to produce an array of health benefits, prompting many providers to recommend the implementation of such activity to those with SCI. Successfully adopting such an exercise regimen however, requires confidence in one's ability to engage in exercise or exercise self-efficacy. Exercise self-efficacy has not been assessed adequately for people with SCI due to a lack of validated and reliable scales, despite self efficacy's status as one of the most widely researched concepts and despite its broad application in health promotion studies. Exercise self efficacy supporting interventions for people with SCI are only meaningful if appropriate measurement tools exist. The objective of our study was to develop a psychometrically sound exercise self-efficacy self-report measure for people with SCI.

**Methods:**

Based on literature reviews, expert comments and cognitive testing, 10 items were included and made up the 4-point Likert SCI Exercise Self-Efficacy Scale (ESES) in its current form. The ESES was administered as part of the first wave of a nationwide survey (n = 368) on exercise behavior and was also tested separately for validity in four groups of individuals with SCI. Reliability and validity testing was performed using SPSS 12.0.

**Results:**

Cronbach's alpha was .9269 for the ESES. High internal consistency was confirmed in split-half (EQ Length Spearman Brown = .8836). Construct validity was determined using principal component factor analysis by correlating the aggregated ESES items with the Generalised Self Efficacy Scale (GSE). We found that all items loaded on one factor only and that there was a statistically significant correlation between Exercise Self-Efficacy Scale (ESES) and Generalised Self Efficacy Scale (GSE) (Spearman RHO = .316; p < .05; n = 53, 2-sided).

**Conclusion:**

Preliminary findings indicate that the ESES is a reliable instrument with high internal consistency and scale integrity. Content validity both in terms of face and construct validity is satisfactory.

## Background

Perceived self-efficacy, defined as *"beliefs in one's capabilities to organize and execute the courses of action required for producing given attainments" *[1], is one of the most widely researched concepts in health promotion. The role of efficacy beliefs in sustaining adherence to exercise regimens has generated some attention in research with the general population [1]. Research has found that people who hold high self efficacy beliefs prior to starting an exercise program show better adherence to an exercise regimen once implemented [2]. This holds true for exercise that is part of a supervised program and personal unsupervised exercise regimens. In short, success in adopting and maintaining regular exercise depends largely on the individual's self-regulatory efficacy.

Persons with spinal cord injury (SCI) have long been ranked at the lowest end of the fitness spectrum [3]. The ever-increasing prevalence of secondary conditions among persons with SCI has focused considerable attention on their poor levels of physical conditioning [4-6]. Notwithstanding the cause, many studies published over the past decades have addressed the need for persons with SCI to adopt habitual exercise as part of a healthy lifestyle [5,7,8]. In many cases the benefits of exercise for those with SCI mirror positive multi-system health benefits documented for those without paralysis, which lend credence to the belief that persons with SCI should avail themselves of habitual exercise insofar as their disability allows.

Self-efficacy is one of the most widely researched concepts with wide application in studies on exercise and other health promotion activities. However, few efforts exist to assess exercise self efficacy in people with SCI. Existing tools are either (a) too generic, (b) not suitable (tasks that cannot be easily performed by people with SCI), (c) too specific (e.g. weight lifting exercise), or (d) have uncertain psychometric properties.

The concept of perceived exercise self-efficacy in people with SCI has found application in research on weight exercise curricula [9]. In this small randomized control study, individuals who received weight exercise instruction paired with self efficacy generalization instruction benefited more from the training in terms of self efficacy than those who only received weight training instructions or a control group without training or self efficacy generalization instruction. Self efficacy generalization beyond one concrete area of application (i.e. weight training) appears to be an important element of health promotion activities, such as exercise curricula. While on the one hand multiple fairly generic, e.g. Generalised Self Efficacy Scale (GSE) [10] and on the other hand highly specific self-efficacy measures exist, (e.g. Weight-Training Efficacy Scale), validated tools that assess exercise self-efficacy with regard to physical activities that people with SCI can conduct in the community, and that are not limited to one specific area of physical activity are lacking. In developing a psychometrically sound tool, we expect that this instrument can find future application in measuring SCI exercise self-efficacy in community-dwelling adults who participate in structured exercise programs or to assess exercise self efficacy beliefs in occasional as well as habitual exercisers with spinal cord injuries.

The objective of our study was to develop the tool and to examine psychometric properties of an exercise self-efficacy self-report measure, called SCI Exercise Self-Efficacy Scale (ESES) for people with spinal cord injury.

## Methods

The study and the informed consent process received approval from the Medstar Research Institute Institutional Review Board (IRB) in Washington, DC.

### Development

#### Literature review

For the purpose of instrument design, we adopted the following working definition for SCI Exercise Self Efficacy: *'SCI Exercise Self Efficacy is one's confidence of individuals with spinal cord injury to plan and carry out physical activities and/or exercise based on their own volition'*.

In generating our item pool, we specifically examined the foundations for exercise self efficacy in the Social Cognition Theory [1]. The initial item pool was generated based on a review of the literature and operationalized in the form of existing instruments. Specifically, we extracted items from the Generalized Perceived Self-Efficacy Scale [10], McAuley's Exercise Self Efficacy Scale for Older People, and the Exercise Benefits/Barrier Scale [11]. A joint pool of 50 items of these scales were reviewed and modified by the research team for presumed appropriateness for the SCI population.

#### Expert review

The scale initial scale produced by the development team consisted of 11 items. Clinical and survey experts were invited to review the scale with regard to item content, clarity, relevance and format. The expert review panel consisted of one physician with spinal cord injury as clinical specialty, two physical therapists, four health service researchers, and two rehabilitation researchers. At the end of the process, one item was dropped and the wording of three items modified.

#### Public review

As the next step, the team invited consumer input from people with spinal cord injury using Internet-based audiocast technology. Listeners were encouraged to provide feedback on content and format of the instrument via e-mail. E-mails were reviewed and suggestions about questions discussed by the research team and incorporated in the item formulation.

#### Cognitive interviews

Cognitive interviews involved six individuals with SCI, five males and one female, aged 31–69 years with annual household incomes ranging from under $20,000 to more than $60,000. Five of the participants were African American, one Caucasian. Three had completed 9–12 years of formal education; two 13–16 years; the information was missing for one participant. Four had complete injuries, two had incomplete injuries. Injury levels varied from cervical spine (C5/6) to lumbar injuries (L2). All six used wheelchairs, three used power, three manual wheelchairs. Three individuals participated in concurrent testing. The interviewer used standardized verbal probes to gather information about how respondents processed the question. Following each individuals response they were then asked how they understood the questions, whether concepts were unclear, what they thought about the format of the questions, and whether they felt that wording or layout were inappropriate. Three individuals participated in retrospective testing of the questionnaire. They were asked to comment on scale instructions, item format, wording, and response options after they completed the ESES.

#### Reliability and validity testing

Based on expert comments and cognitive testing 10 items were included and made up the 4-point Likert SCI Exercise Self-Efficacy Scale (ESES) in its current form. The scale is a self-report measure.

The scale instructs respondents to indicate on the 4-point rating scale (1 = not at all true, 2 = rarely true, 3 = moderately true, 4 = always true) how confident they are with regard to carrying out regular physical activities and exercise. Items are listed in Table 1.

**Table 1:** Internal consistency (alpha) of ESES items.

ESES Items: I am confident	Alpha for Scale .9269 Alpha if Item deleted (n = 368)	Alpha for Scale .8700 Apha if Item deleted (n = 53)	Mean (n = 368)	SD (n = 368)
• that I can overcome barriers and challenges with regard to physical activity and exercise if I try hard enough (ES1)	.9234	.863	3.2582	.8027
• that I can find means and ways to be physically active and exercise (ES2)	. 9153	.850	3.3533	.8450
• that I can accomplish my physical activity and exercise goals that I set (ES3)	.9167	.855	3.1739	.8268
• that when I am confronted with a barrier to physical activity or exercise I can find several solutions to overcome this barrier (ES4)	.9174	.853	3.1359	.8073
• that I can be physically active or exercise even when I am tired (ES5)	.9192	.856	2.8152	.8881
• that I can be physically active or exercise even when I am feeling depressed (ES6)	.9216	.866	2.9918	.9116
• that I can be physically active or exercise even without the support of my family or friends (ES7)	.9189	.851	3.2092	.9666
• that I can be physically active or exercise without the help of a therapist or trainer (ES8)	.9221	.857	3.2989	.9470
• that I can motivate myself to start being physically active or exercising again after I've stopped for a while (ES9)	.9162	.857	3.2880	.8912
• that I can be physically active or exercise even if I had no access to a gym, exercise, training, or rehabilitation facility (ES10)	.9231	.868	3.2446	.9367

Exercise activity was based on recoding of two items that asked whether respondents had exercised at home or outside the home in a gym or both in the past 12 months. The recoded dichotomous item specified 'exercise at home and/or gym' vs. 'no exercise'.

The ESES was administered as part of the first wave of a nationwide survey on exercise behavior in people with SCI in order to reach a large number of respondents with SCI for reliability assessment. Separately, the ESES was presented together with the Generalised Self Efficacy Scale [10] for validity testing to individuals with SCI who participated in four focus groups (16 male; 4 female). Individuals were recruited with the support of the National Spinal Cord Injury Association, local chapters of the organization, the National Rehabilitation Hospital (NRH) in Washington, DC, and the Independent Living Research Utilization (ILRU) in Houston, Texas. Individuals were contacted by e-mail, mail, per telephone or by word-of-mouth.

### Analysis

Reliability of the scale was determined by computing internal consistency coefficient alpha and split-half Spearman Brown for Equal Length.

#### Content validity

Content validity was determined through a series of expert and public reviews and six cognitive interviews with individuals with SCI from diverse demographic and educational backgrounds.

#### Construct validity

Construct validity was determined through Exploratory Principal Component Factor Analysis and by correlating the ESES with the 10-item Generalized Self-Efficacy Scale [10].

Statistical analysis was performed by SPSS version 12.0.

## Results

### Sample characteristics

Internal consistency was determined in a sample of n = 368 individuals with spinal cord injury. Most respondents, 60.1%, were male, and the sample had a mean age of M = 46.29 years (SD = 12.55).

The majority, 85.9%, of the participants were Non-Hispanic White, 7.3% Non-Hispanic Black, with the remaining 6.8% being Hispanic or belonging to another racial/ethnic group. More than half, 53.3%, indicated that they had incomplete injuries.

A smaller subset of participants was involved in the construct validity study (n = 53). The smaller subset was used in order to minimize the respondent burden in the larger survey and to obtain a sufficiently large number of responses on both self-efficacy scales to be able to establish construct validity. Of these, 58.5% were male; the mean age was M = 45.64 years (SD = 13.09). The majority, 77.4%, were Non-Hispanic White, 15.1% Non-Hispanic Black with the remaining 7.6% identifying with another racial or ethnic group. About half, 52.8%, reported that they had incomplete injuries

There were no statistically significant differences between the larger and smaller sample in terms of age, gender, race/ethnicity, marital status or completeness of injury.

### Reliability

We determined the degree of internal consistency for the 10 ESES items by computing Cronbach's alpha separately for the large sample (n = 368) and for the subset that was used to establish construct validity subsequently (n = 53) (see Tables 1, 2). We found a Cronbach's alpha of .9269 for the large sample and of .8700 for the smaller subset. The high values reflect high inter-item correlations and consequently a high degree of internal consistency among items. The item means showed relatively little variation. In our internal consistency analysis for the GSE we found a Cronbach's alpha of .8284 (n = 53).

**Table 2:** Inter-item correlation among items of the SCI Self Efficacy Scale.

	ES1	ES2	ES3	ES4	ES5	ES6	ES7	ES8	ES9	ES10
ES1	1.000									
ES2	.6043	1.000								
ES3	.5891	.7464	1.000							
ES4	.6016	.6883	.7115	1.000						
ES5	.5372	.5738	.6488	.6014	1.000					
ES6	.4646	.5485	.5659	.5309	.6713	1.000				
ES7	.4886	.6065	.5647	.6059	.5023	.5462	1.000			
ES8	.3965	.5657	.4902	.4992	.4644	.4605	.6757	1.000		
ES9	.4824	.6750	.6382	.5855	.6011	.5731	.6194	.5951	1.000	
ES10	.3942	.5722	.4868	.5289	.4869	.4331	.5061	.5778	.6368	1.000

All correlations are significant at the 0.01 level (2-tailed)

### Split-half method

As an alternative to a test-retest approach, we employed split-half internal consistency testing to determine reliability. The reliability of the 10-item scale was .8836 (Equal-Length Spearman-Brown, n = 366). The correlation between the two halves was .7915.

### Content validity

The cognitive interviews, public and expert reviews indicated a good fit of our scale with the concept of self efficacy in relationship to exercise and physical activity.

### Construct validity

An unrotated Principal Component Analysis (n = 366) confirmed that all 10 items loaded on only a single factor, which we call "SCI Exercise Self Efficacy". This factor explained 60.7% of the variance. Item communalities ranged in value between .506 (ES10) and .718 (ES2).

We found further a statistically significant correlation between Exercise Self-Efficacy Scale (ESES) and Generalised Self Efficacy Scale (GSE) (Spearman RHO = .316; p < .05; n = 53, 2-sided). The moderate size of the correlation indicates a good fit with the generic self-efficacy concepts and allows the conclusion that the measure is specific enough that it does not measure the same elements as the generic scale.

### Self-reported exercise behaviour and exercise self efficacy

There were no statistically significant differences between exercisers and non-exercisers in terms of self efficacy summary scores on the Generalised Self Efficacy Scale (GSE) (M = 35.09; SD = 3.3 vs <M = 35.05; SD = 3.2; p > .5) and the Exercise Self Efficacy Scale (ESES) (M = 32.02; SD = 6.9 vs M = 31.27; SD = 6.7; p > .5). However, this may be due to the uneven sample sizes (n = 253 exercisers; n = 113 non-exercisers). We compared the mean ESES scores of exercisers and non-exercisers in a larger sample (n = 396 exercisers; n = 174 non exercisers) and found statistically significant differences between the two groups (p < .001) with higher mean exercise self efficacy scores for exercisers (M = 33.7; SD = 5.3 vs. M = 28.3; SD = 8.3). The latter would indicate that the ESES distinguishes between the two groups. Again, uneven sample sizes do not allow for a definite conclusion.

## Discussion

Preliminary findings indicate that the ESES is a reliable instrument with high internal consistency and scale integrity. Content validity both in terms of face and construct validity is satisfactory. However, study limitations need to be highlighted. While the scale has been developed in several iterative steps and conceptual overlap with a generic self efficacy tool has been demonstrated, it is unclear to what extent the self efficacy tool correlates with measures of exercise and physical activity. Methodologically, reliance on split-half methods to determine the stability of the instrument has been criticized due to the multiple ways the two halves can be formed based on the set of items. Reliability estimates are likely to vary. A future test-retest examination is therefore desirable. The high internal consistency of the scale indicates that fewer items may produce a scale with similar reliability. This is currently explored. The sample for validity testing was much smaller in size, which is a limitation of the study. In our study there does not appear to be a statistically significant difference between self reported exercisers and non exercisers. However, comparisons within a larger sample found significances between the two groups. Unclear is to what extent these findings are attributable to uneven sample sizes. Again, the subsample of n = 53 individuals who completed both self efficacy scales was rather small and may not be representative for the spinal cord injury population at large. Since actual exercise behavior was not recorded it is unclear to what extent self-report truly reflects exercise activity. In the present study exercise behavior was only recorded through self report. The SCI Exercise Self Efficacy Scale needs to be further tested and evaluated in a sample whose physical activity is assessed more comprehensively and rigorously.

Future research is needed to determine the tool's usefulness and sensitivity for capturing change in self-efficacy as a result of community- or home-based exercise programs for this population. As a next step, we will determine test-retest reliability. Recent research has underscored the importance of cognitive-behavioral and motivational factors in promoting exercise after SCI [7].

## Conclusion

Provided further studies support the psychometric soundness of this tool, it will hopefully find wider applicability in clinical and community settings in conjunction with activity- and exercise-focused intervention studies with people after SCI.

## Competing interests

The author(s) declare that they have no competing interests.
